# Inequities in Physical Activity Environments and Leisure-Time Physical Activity in Rural Communities

**DOI:** 10.5888/pcd19.210417

**Published:** 2022-07-07

**Authors:** Michelle C. Kegler, Nicole Gauthreaux, April Hermstad, Kimberly Jacob Arriola, Addison Mickens, Kelley Ditzel, Clarisa Hernandez, Regine Haardörfer

**Affiliations:** 1 Department of Behavioral, Social and Health Education Sciences, Emory Prevention Research Center, Rollins School of Public Health, Emory University, Atlanta, Georgia; 2 Share Health Southeast Georgia, Waycross, Georgia; 3 Public Administration, Georgia College & State University, Milledgeville, Georgia

## Abstract

**Introduction:**

Differential access to environments supportive of physical activity (PA) may help explain racial and socioeconomic disparities in leisure-time physical activity (LTPA) in rural communities.

**Methods:**

We used baseline data from a mailed survey (N = 728) conducted in 2019 as part of an evaluation of The Two Georgias Initiative to examine the relationships among LTPA, sociodemographic characteristics, and perceived access to supportive PA environments (eg, areas around the home/neighborhood, indoor and outdoor exercise areas, town center connectivity) in 3 rural Georgia counties.

**Results:**

More than half of respondents (53.5%) engaged in LTPA in the previous month. Perceptions of PA environments were generally neutral to somewhat negative. In multivariable models, overall PA environment was associated with LTPA (OR, 1.58; 95% CI, 1.06–2.35), as was annual household income >$50,000 relative to ≤$20,000 (OR, 2.72; 95% CI, 1.53–4.83) and race, with Black respondents less likely to engage in LTPA than White respondents (OR, 0.49; 95% CI, 0.29–0.85). Of the 5 PA environment domains examined, town center connectivity was significantly associated with LTPA (OR, 1.68, 95% CI, 1.20–2.36). Both the overall PA score (β = −0.014; 95% CI, −0.029 to −0.002) and town center connectivity (β = −0.020; 95% CI, −0.038 to −0.005) partially mediated associations between annual household income and LTPA. Areas supportive of PA around the home/neighborhood partially mediated the association by race (β = 0.016; 95% CI, 0.001–0.034).

**Conclusion:**

Findings lend support for investing in town centers and racially diverse neighborhoods to increase walkability and PA infrastructure as potential strategies to reduce inequities in LTPA.

**Summary**

**What is already known on this topic?**

Disparities in physical activity exist by race, socioeconomic status, and rurality. Many rural areas have limited resources for leisure-time physical activity.

**What is added by this report?**

Access to physical activity resources, especially town center connectivity, was associated with leisure-time physical activity. Disparities in leisure-time physical activity were partially explained by perceptions of town center connectivity for lower-income residents and by perceptions of areas around the home/neighborhood for Black residents.

**What are the implications for public health practice?**

Investments in town center connectivity and racially diverse neighborhoods may aid in reducing inequities in leisure-time physical activity among rural residents.

## Introduction

Rural residents are less likely to meet federal physical activity (PA) guidelines than their urban counterparts, with the lowest rates among rural residents in the South (1–3). Although rural communities vary, they typically have lower levels of educational attainment, higher levels of poverty, less investment in infrastructure, and distance and/or terrain that decreases access to health-promoting resources (4,5). Scholars have urged the consideration of rurality in intersectionality approaches that examine how overlapping social categories produce inequalities based on disadvantaged social position (6–9). Knowledge about intersections of rurality, race, and socioeconomic status (SES) help to illuminate disparities in PA (1,2,10). According to the 2016–2017 National Health Interview Survey, 17.9% of rural Black adults met PA guidelines, compared with 27.8% of urban White adults, and a similar disparity was observed for rurality and education (ie, lowest rates of PA for rural residents with less education) (1).

Although PA is influenced by an array of determinants, studies generally show associations between features of the environment that support active living (eg, streetlights, paths, access to recreational facilities, aesthetics) and PA across the rural–urban continuum (11–14). Consistent with structural models that highlight the interplay among physical structures, social structures, and policies in producing inequities (15), using an equity lens necessitates a deeper examination of who has access to environments that support PA and whether differential access to health-promoting environments can explain differences in PA by race and SES in rural areas.

Given the possibility that unequal access to supportive environments may contribute to disparities in PA (7,13,16), the aims of this study were to examine 1) associations between the dimension of the PA environment and leisure-time PA (LTPA), 2) differential access to PA environments by race, SES, and neighborhood rurality, and 3) whether environments mediate relationships between race, SES, and LTPA in rural communities.

## Methods

The Two Georgias Initiative is a place-based initiative designed to achieve greater health equity among rural Georgians. In 2017, the Healthcare Georgia Foundation funded 11 coalitions in rural Georgia (county population <35,000) to conduct a community assessment, develop a community health improvement plan, and implement strategies to address a range of community-identified priority areas, including efforts to improve PA environments. Throughout the 5-year initiative, coalitions received up to $100,000 per year and technical assistance and support from a community coach, external evaluators, and equity experts.

### Data collection

This cross-sectional study used data from a baseline population-based survey that explored behaviors and environments related to common coalition priority areas. The survey was designed with different modules that mapped to local priorities: PA and access to active living environments, healthy eating and food access, health care access, social capital, and quality of life. Respondents were included in the study if they lived in 1 of the 3 counties in which the full PA module was administered (N = 728). In 2018, the population sizes in these counties ranged from 5,800 to 18,500, and Rural-Urban Continuum Codes were 7 and 8 (17).

We used commercial lists of randomly selected residential mailing addresses to identify the sampling frame, which varied by county size. One adult per household was eligible to participate. Baseline surveys were mailed in waves from January through March 2019. Each mailing included an introductory letter describing the study and information on the incentive (a $15 gift card), the survey, and a stamped return envelope. Each household received a postcard reminder and, if necessary, a second survey. A total of 2,671 surveys were sent, and 728 were completed (27.2% response rate); the county-specific response rate ranged from 25.6% to 31.7%. The Emory University Institutional Review Board determined that this study was a nonresearch program evaluation that did not require institutional review board approval.

### Measures

#### Leisure-time physical activity

We used a measure from the Centers for Disease Control and Prevention’s Behavioral Risk Factor Surveillance System (BRFSS) to measure LTPA (18). Respondents were asked, “During the past month, other than your regular job, did you participate in any physical activities or exercises such as running, calisthenics, golf, gardening, or walking for exercise?” Response options were yes, no, or don’t know/not sure. We excluded responses of don’t know/not sure from analyses that used this variable.

#### Walking and biking for transport

We assessed walking and biking for transport by asking, “How often do you use each of the following to get from place to place?” and then listing walk, bike, personal vehicle (car/truck/sport utility vehicle), golf cart, ride from family/friend/neighbor, taxi service or rideshare such as Uber/Lyft, and public transportation (bus/van). Response options ranged from 0 (never) to 4 (daily) (19).

#### Perceived PA environments

Questions on overall PA environment and 5 domains were adapted from the Rural Active Living Perceived Environmental Support Scale, or RALPESS (20). We chose 22 items from the original measure. We computed a mean score for each of 5 domains by averaging the scores obtained from a 5-point Likert scale, where 0 indicated strongly disagree; 1, disagree; 2, neutral; 3, agree; and 4, strongly agree. We computed an overall score by averaging all 22 items, with selected items reverse coded as necessary. Cronbach α was 0.85.

*Area around your home/neighborhood* contained 6 items: 1) the roads around my home have a place to walk or ride a bike next to the road, 2) the roads around my home have good lighting, 3) it is safe to walk or ride a bike on the roads around my home, 4) there is fast traffic on the roads around my home, 5) there are busy roads to cross around my home, and 6) loose dogs in the area around my home make it unsafe to take walks. Cronbach α was 0.67.

*Indoor exercise area*s contained 3 items: 1) my town has private indoor exercise areas (pay to use), 2) my town offers indoor exercise activities (eg, programs, sports teams, classes, lessons, etc), and 3) there are choices of activities for PA or exercise at the indoor exercise areas in my town. Cronbach α was 0.87.

*Outdoor exercise areas* contained 4 items: 1) outdoor exercise areas in my town have available restrooms, 2) outdoor exercise areas in my town have water fountains, 3) outdoor exercise areas are nice to use, and 4) police officers or sheriffs regularly patrol the outdoor areas in my town where people could be physically active or exercise. Cronbach α was 0.88.

*Town center connectivity* contained 5 items: 1) there are shopping areas and places to eat in the town center, 2) there are sidewalks in the town center, 3) the sidewalks are nice to use in the town center (eg, they are shaded, there are pleasant things to look at, no trash, well kept), 4) the streets are marked where I should cross in the town center or there are crosswalks, and 5) the area around the town center has working streetlights. Internal consistency was good for this domain (Cronbach α = 0.88)

Lastly, *school and church facilities* were combined to create an *organizational facilities* domain of 4 items: 1) at least 1 school allows community members to use *indoor* facilities during nonschool hours, 2) at least 1 school allows community members to use *outdoor* facilities during nonschool hours, 3) my community has churches with *indoor* recreational areas for exercise open to the public, and 4) my community has churches with *outdoor* recreational areas for exercise open to the public. Cronbach α was 0.69.

#### Sociodemographic characteristics

The 3 primary sociodemographic variables of interest were race, annual household income, and neighborhood rurality. Race and ethnicity were assessed by asking, “What is your race or ethnicity?” Response options were White, not of Hispanic origin; African American or Black, not of Hispanic origin; Hispanic; more than 1 race; and other. We dichotomized into White or Black, with other races and ethnicities excluded in multivariable models and bivariate analyses examining racial differences because of small numbers. Annual household income was assessed by asking, “What is your total yearly household or family income from all sources?” Response options included 3 categories, which we combined according to distribution: $20,000 or less, $20,001 to $50,000, and more than $50,000. Neighborhood rurality was assessed with the question, “Which of the following best describes the neighborhood where you live? By neighborhood, we mean the area within a 20-minute walk from your home.” This item was created by our team to capture qualitative differences based on where one lived in a rural county. We combined responses into 2 categories: in town or rural area. The survey also assessed age, sex, education, employment, marital status, and height and weight, which were used to calculate body mass index (BMI).

### Data analysis

We used SPSS version 26.0 (IBM Corp) and SAS version 9.4 (SAS Institute, Inc) to conduct descriptive and multivariable analyses. Sociodemographic differences between those engaged in LTPA and those who were not were assessed by using *t* tests, analysis of variance, and χ^2^ tests, as appropriate for each variable type. Differences in PA environments by group (eg, race) were tested by using 1-way analysis of variance and *t* tests, with the Tukey honestly significant difference test used to identify posthoc differences between groups. We used multivariable logistic regressions to assess associations between LTPA and the PA environment. The first model included the overall PA environment score and the second included the individual domain scores; both models included race, annual household income, and neighborhood rurality as independent variables, while controlling for sex, age, and county. We excluded records with data missing on any of these variables (ie, we used listwise deletion).

To explore whether PA environments may at least partly explain racial differences in LTPA, we used structural equation modeling through variable-measured path models with 1 mediator, which is equivalent to a simple mediation model that accommodates noncontinuously distributed variables and allows for clustering (21). At the bivariate level, we conducted simple linear regression models between the independent variables and each of the 5 PA environment domains and overall score. We used MPlus version 8.4 (Muthen & Muthen) to conduct mediation analyses to assess whether the PA environment (ie, overall and specific domains) mediates the effects of race or income on PA. Models accounted for clustering in counties, and 95% CIs for the indirect effects were determined by using bootstrapping (n = 1,000).

## Results

Overall, the sample was 68.9% female and 32.4% Black, with a mean age of 60.5 (SD, 15.4) years (Table 1). Respondents were well distributed across annual household income categories: 30.0% lived on $20,000 or less, 38.4% on $20,001 to $50,000, and 31.7% on more than $50,000. Similarly, we found a broad range of education levels and employment: 41.1% were retired and 34.8% worked full-time. More than half (58.1%) were married or living with a partner. Most described themselves as living in rural areas of the county (81.1%) as opposed to in town (18.9%).

**Table 1:** Description of Survey Respondents (N = 728), by Leisure-Time Physical Activity Status, in 3 Rural Counties in Georgia, 2019^a^

Characteristic	Full sample (N = 728)^b^	Engaged in leisure-time PA in past month (n = 348)^b^	Did not engage in leisure-time PA in past month (n = 303)^b^	*P* value^c^
**Age, mean (SD), y**	60.5 (15.4)	57.9 (15.1)	63.4 (15.0)	<.001
**Sex**				
Male	217 (31.1)	115 (58.1)	83 (41.9)	.17
Female	480 (68.9)	228 (52.2)	209 (47.8)	
**Race^d^**				
White	456 (67.6)	250 (59.1)	173 (40.9)	<.001
Black	219 (32.4)	82 (42.5)	111 (57.5)	
**Annual household income, $**				
≤20,000	175 (30.0)	58 (38.4)	93 (61.6)	<.001
20,001–50,000	224 (38.4)	109 (53.2)	96 (46.8)	
>50,000	185 (31.7)	122 (70.9)	50 (29.1)	
**Education**				
Some high school or less	81 (11.5)	27 (38.6)	43 (61.4)	<.001
High school diploma/GED	207 (29.3)	80 (42.6)	108 (57.4)	
Some college or technical school	221 (31.1)	111 (55.8)	88 (44.2)	
College and above	197 (27.9)	126 (69.6)	55 (30.4)	
**Employment**				
Working full-time	237 (34.8)	138 (62.4)	83 (37.6)	<.001
Working part-time	45 (6.6)	22 (53.7)	19 (46.3)	
Retired	280 (41.1)	133 (53.4)	116 (46.6)	
Not employed/homemaker/student/on disability	120 (17.6)	39 (35.8)	70 (64.2)	
**Marital status**				
Married/living with partner	423 (58.1)	224 (58.8)	157 (41.2)	.002
Separated/divorced/widowed	201 (27.6)	102 (42.7)	76 (57.3)	
Single	104 (14.3)	48 (52.1)	44 (47.8)	
**Neighborhood rurality**				
In town	120 (18.9)	50 (41.7)	70 (58.3)	.002
Rural	514 (81.1)	293 (57.0)	221 (43.0)	
**County of residence**				
County A	276 (37.9)	131 (52.8)	117 (47.2)	.19
County B	242 (33.2)	121 (58.2)	87 (41.8)	
County C	210 (28.8)	96 (49.2)	99 (50.8)	
**Active transportation modes, mean (SD)^e^**				
Walking	1.4 (1.5)	1.7 (1.5)	1.1 (1.4)	<.001
Biking	0.3 (0.8)	0.4 (0.8)	0.2 (0.8)	.04
**Body mass index, kg/m^2^**				
Normal weight (18.5–24.9)	158 (23.4)	89 (62.7)	53 (37.3)	<.001
Overweight (25.0–29.9)	226 (33.5)	125 (61.6)	78 (38.4)	
Obese (≥30.0)	291 (43.1)	115 (43.7)	148 (56.3)	
**Physical activity environment domains, mean (SD)^f^**				
Around home/neighborhood	1.6 (0.8)	1.7 (0.9)	1.6 (0.8)	.17
Indoor exercise areas	2.0 (1.2)	2.1 (1.2)	1.9 (1.2)	.11
Outdoor exercise areas	1.7 (1.0)	1.7 (1.0)	1.7 (1.0)	.32
Town center connectivity	2.5 (0.8)	2.6 (0.7)	2.4 (0.8)	.001
School and church facilities	1.7 (0.8)	1.7 (0.7)	1.6 (0.8)	.24
Overall composite score	1.9 (0.6)	2.0 (0.03)	1.8 (0.03)	.02

Abbreviations: GED, General Educational Development.

a The authors developed and administered a baseline population-based survey that explored behaviors and environments related to physical activity. All values are number (percentage) unless otherwise indicated; percentages may not add to 100 because of rounding.

b Numbers in categories may not add to numbers in column headers because not all respondents answered all questions; percentages are based on the number of respondents who answered the question. Responses of don’t know/not sure were excluded.

c For continuous variables (mean [SD]), *t* test was used. For categorical variables, χ^2^ test was used.

d Assessed by asking, “What is your race or ethnicity?” Response options were White, not of Hispanic origin; African American or Black, not of Hispanic origin; Hispanic; more than 1 race; and other. Responses were dichotomized into White or Black, with other races and ethnicities excluded in multivariable models and bivariate analyses examining racial differences because of small numbers.

e Assessed by asking, “How often do you use each of the following to get from place to place?” and then listing walk, bike, personal vehicle (car/truck/sport utility vehicle), golf cart, ride from family/friend/neighbor, taxi service or rideshare such as Uber/Lyft, and public transportation (bus/van). Response options ranged from 0 (never) to 4 (daily).

f Overall physical activity environment and specific domains were adapted from the Rural Active Living Perceived Environmental Support Scale, or RALPESS (20); 22 items were chosen from the original measure. A mean score for each domain was computed by averaging the scores obtained from a 5-point Likert scale, where 0 indicated strongly disagree; 1, disagree; 2, neutral; 3, agree; and 4, strongly agree. An overall score was computed by averaging all 22 items, with selected items reverse coded as necessary.

### LTPA by sociodemographic characteristics

Overall, slightly more than half (53.5%; 348 of 651) of respondents had engaged in LTPA at least once in the previous month. We found significant differences in LTPA for every variable we examined except sex and county (Table 1). LTPA was most common among White respondents (59.1%), those with annual household income of more than $50,000 (70.9%), those with a college degree (69.6%), and those who worked full-time (62.4%). Respondents engaging in LTPA were younger on average, more frequently walked or biked for transport, and were normal weight (62.7%) or overweight (61.6%). Those living in rural areas (57.0%) were more likely to engage in LTPA than those living in town (41.7%).

### Physical activity environments by LTPA

The composite PA environment score was associated with LTPA for respondents who engaged in LTPA (mean, 2.0; SD, 0.03) versus those who did not (mean, 1.8; SD, 0.03) (Table 1). Only 1 domain, town center connectivity, was associated with LTPA (mean, 2.6; SD, 0.7 vs mean, 2.4; SD, 0.8). Although significant, differences were small for both the overall composite score and the town center connectivity domain. Indoor exercise areas, outdoor exercise areas, and school and church facilities were not associated with LTPA.

### Physical activity environments by selected demographic and neighborhood variables

Ratings of the PA environments generally ranged from neutral to slightly negative (range, 1.4–2.6) (Table 2). On the basis of the overall composite score, respondents with higher annual household incomes perceived their PA environments slightly more positively than respondents with middle and low household incomes. Black respondents viewed their PA environments slightly more negatively than White respondents, and we found no significant difference based on neighborhood rurality.

**Table 2:** Physical Activity Environments, by the 3 Primary Sociodemographic Variables of Interest, in the Full Sample of Survey Respondents (N = 728) in 3 Rural Counties in Georgia, 2019^a^

Characteristic	Around home or neighborhood	Indoor exercise areas	Outdoor exercise areas	Town center connectivity	School and church facilities	Overall composite score
**Rating by neighborhood rurality, mean (SD)**						
In town	1.8 (0.8)	2.1 (1.2)	1.8 (1.0)	2.5 (0.7)	1.7 (0.8)	2.0 (0.5)
Rural	1.6 (0.8)	2.0 (1.7)	1.7 (1.0)	2.5 (0.8)	1.7 (0.8)	1.9 (0.6)
*P* value^b^	.009	.35	.34	.64	.41	.07
**Rating by annual household income, mean (SD), $**						
≤20,000	1.7 (0.7)	1.8 (1.2)	1.6 (1.0)	2.3 (0.8)	1.6 (0.8)	1.8 (0.6)
20,001–50,000	1.6 (0.8)	1.8 (1.2)	1.6 (1.0)	2.5 (0.8)	1.6 (0.8)	1.8 (0.6)
>50,000	1.7 (0.9)	2.3 (1.2)^c^	1.9 (0.9)^c^	2.6 (0.7)^d^	1.8 (0.7)	2.0 (0.6)^c^
*P* value^e^	.76	<.001	.005	.004	.05	.003
**Rating by race, mean (SD)**						
Black	1.9 (0.8)	1.7 (1.2)	1.4 (1.0)	2.3 (0.8)	1.5 (0.9)	1.8 (0.6)
White	1.6 (0.8)	2.1 (1.2)	1.9 (0.9)	2.6 (0.7)	1.7 (0.7)	2.0 (0.6)
*P* value^b^	<.001	<.001	<.001	<.001	.001	.003

a The authors developed and administered a baseline population-based survey that explored behaviors and environments related to physical activity. Questions on overall physical activity environment and 5 domains were adapted from the Rural Active Living Perceived Environmental Support Scale, or RALPESS (20). The survey asked respondents to respond to such statements as “The roads around my home have good lighting” and “Outdoor exercise areas in my town have water fountains.” Response options were 0, strongly disagree; 1, disagree; 2, neutral; 3, agree; and 4, strongly agree; missing data were excluded from denominators.

b Determined by *t* test.

c Households with >$50,000 in annual household income had significantly more positive perceptions than respondents in middle- and low-income groups.

d Households with >$50,000 in annual household income had significantly more positive perceptions than respondents in the lowest income groups.

e Determined by analysis of variance, with Tukey honestly significant difference test.

Three of the 5 PA environment domains differed by annual household income: indoor exercise areas, outdoor exercise areas, and town center connectivity (Table 2). For 2 domains (indoor and outdoor exercise areas), respondents with an annual household income of more than $50,000 had more positive perceptions than respondents in the 2 lower income categories. For town center connectivity, respondents with annual household incomes of more than $50,000 had more positive perceptions than those with annual household incomes of $20,000 or less.

All 5 PA environment domains differed by race (Table 2). White respondents had more positive perceptions than Black respondents about indoor exercise areas, outdoor exercise areas, town center connectivity, and school and church facilities. Black respondents, in contrast, had more positive perceptions about the area around their homes. Only 1 PA environment domain differed by neighborhood rurality: respondents living in town viewed the area around their home more positively than did those who lived in more rural areas.

### Multivariable associations between sociodemographic characteristics, PA environments, and LTPA

In the first model, the overall composite PA environments score was significantly associated with engaging in any LTPA (OR, 1.58; 95% CI, 1.06–2.35) (Table 3). Annual household income of more than $50,000 was also associated with increased odds of LTPA (OR, 2.72; 95% CI, 1.53–4.83). Black respondents were less likely than White respondents to engage in LTPA (OR, 0.49; 95% CI, 0.29–0.85).

**Table 3:** Multivariable Logistic Regression Models of Associations Between Leisure-Time Physical Activity and Physical Activity Environments in 3 Rural Counties in Georgia, 2019

Model	Odds Ratio (95% CI)
**Model 1** a **: overall physical activity environment**	
Composite physical activity environment score	1.58 (1.06–2.35)
Rural (vs in town)	1.33 (0.79–2.44)
Black (vs White)	0.49 (0.29–0.85)
Annual household income $20,001 to $50,000 (vs ≤$20,000)	1.66 (1.00–2.76)
Annual household income >$50,000 (vs ≤$20,000)	2.72 (1.53–4.83)
**Model 2** a **: physical activity environment components**	
Town center	1.68 (1.20–2.36)
Around home	1.10 (0.84–1.44)
Indoor areas in town	0.88 (0.70–1.11)
Outdoor areas in town	0.99 (0.74–1.33)
School and church facilities	1.09 (0.80–1.48)
Rural (vs in town)	1.28 (0.75–2.17)
Black (vs White)	0.51 (0.29–0.88)
Annual household income $20,001 to $50,000 (vs ≤$20,000)	1.60 (0.95–2.68)
Annual household income >$50,000 (vs ≤$20,000)	2.83 (1.57–5.07)

a The authors developed and administered a baseline population-based survey that explored behaviors and environments related to physical activity. Questions on overall physical activity environment and 5 domains were adapted from the Rural Active Living Perceived Environmental Support Scale, or RALPESS (20). The survey asked respondents to respond to such statements as “the roads around my home have good lighting” and “outdoor exercise areas in my town have water fountains.” N = 439 (analysis did not include respondents with missing values); adjusted for covariates (county, sex, and age). The 3 primary sociodemographic variables of interest were race, annual household income, and neighborhood rurality.

In the second model, of the 5 dimensions examined, only perceptions of town center connectivity was significantly associated with LTPA (OR, 1.68; 95% CI, 1.20–2.36). Race and annual household income were also significant, with Black respondents less likely than White respondents (OR, 0.51; 95% CI, 0.29–0.88) and respondents with an annual household income of more than $50,000 more likely to engage in LTPA (OR, 2.83; 95% CI, 1.57–5.07).

### Mediation analyses

**Figure 1:**
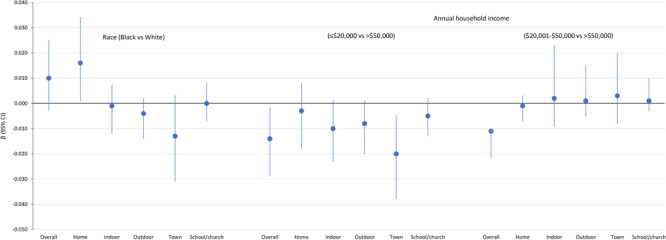
Standardized mediation effect sizes estimating the indirect effect for the overall score on the physical activity environment and component scores as mediators between race or annual household income and physical activity in 3 rural counties in Georgia, 2019.

In models assessing whether perceptions of the PA environment mediate the relationship between annual household income and PA, we found that the composite PA environment score mediated this relationship for respondents in the lowest income category (β = −0.014; 95% CI, −0.029 to −0.002) and middle income category (β = −0.011; 95% CI, −0.022 to −0.011) compared with respondents in the highest income category (Figure 1). Of the 5 domains (Figure 1), only town center connectivity significantly mediated the relationship between low annual household income and PA (β = −0.020; 95% CI, −0.038 to −0.005).

**Table:** 

Relationship	Indirect effect, beta (95% CI)
**Race on physical activity**	
Overall score	0.010 (−0.003 to 0.025)
Home	0.016 (0.001 to 0.034)
Indoor	−0.001 (−0.012 to 0.007)
Outdoor	−0.004 (−0.014 to 0.002)
Town	−0.013 (−0.031 to 0.003)
School/church	0 (−0.007 to 0.008)
**Low income on physical activity**	
Overall score	−0.014 (−0.029 to −0.002)
Home	−0.003 (−0.018 to 0.008)
Indoor	−0.010 (−0.023 to 0.001)
Outdoor	−0.008 (−0.020 to 0.001)
Town	−0.020 (−0.038 to −0.005)
School/church	−0.005 (−0.013 to 0.002)
**Low/medium income on physical activity**	
Overall score	−0.011 (−0.022 to −0.011)
Home	−0.001 (−0.007 to 0.003)
Indoor	0.002 (0.023 to −0.009)
Outdoor	0.001 (0.015 to −0.005)
Town	0.003 (0.020 to −0.008)
School/church	0.001 (0.010 to −0.003)

**Figure 2:**
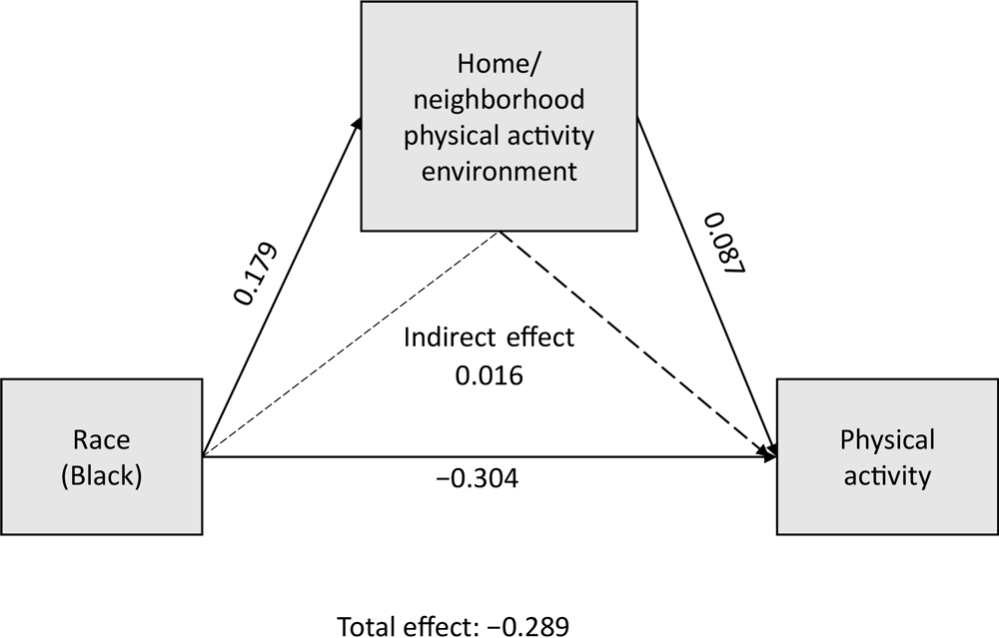
Home/neighborhood physical activity environment as a mediator of the relationship between race and leisure-time physical activity in 3 rural counties in Georgia, 2019.

In our assessment of whether perceptions of the PA environment mediate the relationship between race and LTPA, we found that the overall score did not (Figure 1), but the home/neighborhood environment partially mediated the relationship between race and LTPA (β = 0.016; 95% CI, 0.001–0.034) (Figure 1 and Figure 2).

## Discussion

We examined whether perceived access to environments supportive of PA was associated with increased LTPA. We also examined access to these environments by race, SES (annual household income), and neighborhood rurality, and whether differential access to supportive environments may explain racial and SES differences in LTPA in a rural population. In multivariable analyses, we found that residents in these rural counties who reported a more favorable PA environment were more likely to engage in LTPA. Although most studies of the environment and PA have focused on urban and suburban environments, several studies have documented associations in rural populations, albeit with varied dimensions emerging as salient (12,13,22–24). Of the 5 PA environment domains we examined, town center connectivity was significantly associated with LTPA in multivariable models. Other domains, including one’s home/neighborhood, indoor and outdoor exercise areas, and school and church facilities were not significantly associated with LTPA. This finding may have resulted partly from a measurement issue for the home/neighborhood domain. Previous qualitative research in the same region found that much PA around the home occurred through yard work, which would not have been captured in the home/neighborhood measure because of its focus on walkability (25). Notably, perceptions of the various domains were mainly neutral to negative across the board. This limited variability may have reduced our ability to detect associations with LTPA. Previous research on these domains in the RALPESS instrument focused on validity and reliability of the measure, excluding predictive validity (20,26).

We also examined perceptions of access to various PA domains. Our findings generally support past research that documents that supportive PA environments are less available in rural areas than in nonrural areas (24,27,28). Only the area around one’s home varied by neighborhood rurality; however, all domains varied by race and most also varied by annual household income. In general, White respondents reported better access than Black respondents in all domains except around one’s home. Overall PA environment and 3 domains varied by annual household income, including indoor and outdoor recreation areas and town center connectivity.

Our study also examined whether differential access to environments supportive of PA might explain racial and SES differences in PA during leisure time. Results showed that the overall PA environment partially mediated the association between annual household income and LTPA. This finding lends support to investing in PA environments as a strategy to promote PA and reduce income-related inequities. To our knowledge, ours is one of the first studies to examine how PA environments may mediate associations between race and LTPA in rural communities (15). In an examination of which domains may mediate this association, town center connectivity was significant. These findings, even though the effect was small, lend support to main street investment that increases walkability (eg, sidewalks, lighting, aesthetics, destinations) and other efforts to improve access to PA opportunities given their potential to increase LTPA in rural communities, especially for lower-income households. Several community coalitions in The Two Georgias Initiative are working to improve PA infrastructure (eg, playgrounds, walking trails) and stimulate new destinations in the town center (eg, walking tour of historic sites, flea markets).

We found that the area around one’s home partially mediated the association between race and LTPA. Specifically, Black respondents reported slightly more positive walkability around their homes (eg, safe to walk/ride bike on roads), and this factor reduced the association between race and LTPA. Although the effect was small and based on cross-sectional data, this finding suggests that efforts to increase neighborhood walkability, especially in majority-Black neighborhoods, deserve attention as a potential strategy to increase LTPA. A review of the built environment and PA among African Americans noted some evidence for sidewalks, light traffic, and safety from crime as associated with PA (29). Not having time or transportation to gain access to spaces for PA has also been identified as a barrier for African Americans (30) and provides further justification for a neighborhood focus. Moreover, given the history of redlining, residential segregation, and disinvestment in Black neighborhoods that has resulted in neighborhoods with fewer health-promoting amenities (16,31,32), this approach would be an appropriate step toward health equity.

### Limitations

This study has several limitations. First, it was cross-sectional, which negates our ability to infer causality. It may be that people who engage in LTPA view their environments as more conducive to PA based on their direct experience in these environments. Second, our measures, including PA, were self-reported and may have been subject to social desirability bias. Additionally, it would have been useful to have complementary objective measures of the environment. Third, although the sample was randomly selected, the response rate was low and respondents tended to be older, more educated, and White relative to the overall population in the US census. Despite this, our response rate and methods are consistent with recent recommendations (and response rates) for surveillance in rural communities (33). Fourth, even though we adjusted for clustering at the county level, clustering within smaller geographic units may have occurred. Fifth, we modified the RALPESS instrument to align with evaluation objectives, which may have decreased its reliability. Finally, we focused on LTPA. Thus, our findings do not apply to all forms of PA, including occupational and transport-related activity.

### Conclusion

These findings, even though the effect was small and data were cross-sectional, lend support to downtown improvement projects that increase walkability (eg, sidewalks, crosswalks, lighting, aesthetics, destinations) and other efforts to improve access to PA opportunities given their potential to increase LTPA in rural communities, especially for lower-income households. Our study also suggests that investing in Black neighborhoods to make them more supportive of PA (eg, improved sidewalks, lighting, nice aesthetics, destinations) may pay dividends in terms of public health outcomes such as increased PA. Overall, this study adds to the limited research on PA in rural areas and provides insight into whether and how the environment may contribute to racial and SES differences in LTPA.
